# Diagnostics of Gonococcal Infection in Ukraine: Current Challenges in Resource-Constrained Settings

**DOI:** 10.5152/eurasianjmed.2021.20043

**Published:** 2021-10

**Authors:** Iryna Boiko, Inna Krynytska, Ihor Kohut, Halyna Bezkorovaina, Viktor Stepanenko

**Affiliations:** 1 Department of Functional and Laboratory Diagnostics, I.Horbachevsky Ternopil National Medical University, Ternopil, Ukraine; 2 Department of Infectious Diseases with Epidemiology, Skin and Sexually Transmitted Diseases, I.Horbachevsky Ternopil National Medical University, Ternopil, Ukraine; 3 Department of Pathophysiology, I.Horbachevsky Ternopil National Medical University, Ternopil, Ukraine; 4 Department of Dermatology and Venereology, Bogomolets National Medical University, Kyiv, Ukraine

**Keywords:** Neisseria gonorrhoeae, Gonorrhoea, diagnostics, culture, Ukraine

## Abstract

**Objective:**

This study aimed to evaluate the compliance of laboratory diagnostics of gonorrhoea in Ukraine with the World Health Organization (WHO) laboratory manual.

**Materials and Methods:**

A quantitative non-randomised cross-sectional descriptive postal survey was conducted to evaluate the diagnostics of gonorrhoea in sexually transmitted infections (STI) clinics in Ukraine.

**Results:**

The survey provided data about diagnostics of *Neisseria gonorrhoeae* in STI clinics from 14 regions of Ukraine from January 2013 to September 2014. The clinics performed microscopy, culture, and point-of-care-testing in 100%, 85.7%, and 7.1% of the cases, respectively. None of the respondents had the option of performing nucleic acid amplification tests and antimicrobial susceptibility testing. Two regions reported their participation in the collaborative project by WHO on antimicrobial resistance investigation, as national antimicrobial susceptibility surveillance program had not been established in Ukraine. A “three-site testing” (urogenital, pharyngeal, and rectal specimens) in symptomatic heterosexuals was conducted by 25%, “two-site testing” (urogenital and pharyngeal specimens) was conducted by 41.7%, and “one-site testing” (urogenital specimen) was conducted by 33.3% of the respondents. External quality control of laboratory tests for the detection of *N. gonorrhoeae* was not performed in 50% of the regions in Ukraine. Non-selective culture media for isolation of *N. gonorrhoeae* and culture media in tubes instead of the recommended Petri dishes were used in 16.7% and 58.3% of the laboratories, respectively.

**Conclusion:**

Increased adherence to evidence-based WHO and/or nationally adapted management guidelines is essential for monitoring gonorrhoea and preventing antimicrobial resistance of *N. gonorrhoeae* in Ukraine.

## Introduction

Gonorrhoea is a significant public health problem globally due to its severe complications related to reproductive and sexual health.^1-^^4^ According to the World Health Organization (WHO), the worldwide estimated incidence of gonorrhoea in adults was 86.9 million cases in 2016.^5^ Nevertheless, reported rates of gonorrhoea vary considerably.^6^ The incidence of gonorrhoea in Ukraine showed a 2.8-fold decrease from 2008 to 2018 and an incidence of 9.7 cases per 100,000 individuals was reported in 2018.^7^ The prevalence of gonorrhoea in many Eastern-European countries is underestimated due to suboptimal diagnostics, case reporting, and surveillance.^5,8–12^ It is a matter of grave concern that *N. gonorrhoeae* has developed antimicrobial resistance (AMR) to all drugs administered for the treatment of gonorrhoea.^1,8,13–15^

Responsive and reliable management is crucial for controlling gonococcal infection.^1,8,14,16^ Evidence-based international and/or national guidelines for the management of gonorrhoea are essential^17–20^ and it is necessary to monitor the adherence of diagnostic practice to these guidelines.^21–24^ In 2013, the WHO issued the manual named “Laboratory diagnosis of sexually transmitted infections, including human immunodeficiency virus” (WHO laboratory manual), which reflects the best international experience in laboratory diagnosis of gonorrhoea.^17^ Ukrainian laboratory guideline for sexually transmitted infections (STI) has not been updated for the last 20 years,^25^ which is a matter of concern. Pilot evaluation of laboratory diagnosis of gonorrhoea was performed in three out of 27 regions of Ukraine in 2013.^26^ However, extended assessment of *N. gonorrhoeae* laboratory management according to WHO standard has not been conducted in Ukraine.

In the present study, we aimed to evaluate the compliance of laboratory diagnostics of gonorrhoea in Ukraine with the WHO laboratory manual and to provide data for updating the national laboratory guideline.

## Materials and Methods

### Study Design

A quantitative non-randomised cross-sectional descriptive postal survey of gonorrhoea laboratory diagnostics was performed in 27 specialised and leading government-funded public regional STI clinics across Ukraine from November 2014 to February 2015. The survey included statistical data, close-ended questions regarding the laboratory methods for the detection of *N. gonorrhoeae*, and details about culture methods from January 2013 to September 2014. The questionnaire was pre-tested before sending it to the participants. Respondents replied by e-mails or postal letters. No patient information was disclosed.

### Respondents of Gonorrhoea Diagnosis Survey in Ukraine

Respondents from 14 (14/27, 51.9%) STI clinics replied to the survey questionnaire regarding laboratory methods used for the detection of *N. gonorrhoeae*. Respondents from 12 STI clinics (12/27, 44.4%) replied to the questionnaire regarding gonococcal culturing and the anatomical sites preferred for collecting the specimens. Respondents represented different regions of Ukraine. A population of more than 200,000 was under the supervision of every STI clinic included in the survey (Table 1).

Each STI clinic had a laboratory department where all diagnostics and identifications tests were run. The positive detection rates of *N. gonorrhoeae* were collected from the respondents as proof of their access to gonorrhoea patients. In general, *N*. *gonorrhoeae* was detected by both microscopy and culture in 2.3% (1740/76,348 and 1322/56,273, respectively) of the tested patients (Table 2).

### Statistical Analysis

Thematic analysis was performed to evaluate the answers of the respondents. Calculation of 95% confidence interval was performed using the exact binominal distribution method. All statistical analyses were performed using the MedCalc Statistical Software version 18.11.3 (MedCalc Software bvba, Ostend, Belgium).

### Ethical Approval

Bioethics Commission of I. Horbachevsky Ternopil National Medical University of the Ministry of Health of Ukraine approved the study (Excerpts from Minutes No. 29, dated 20.05.2015).

## Results

The answers regarding laboratory methods of *N. gonorrhoeae* detection are summarised in Table 3. All respondents performed microscopy, culture for *N. gonorrhoeae*, and point-of-care-test (POCT) using the “Gono-test-MBA” immunochromatographic test kit for the detection of *N. gonorrhoeae* (MedBio Alliance, Kyiv, Ukraine) in 100%, 85.7%, and 7.1% of the cases, respectively. None of the regional STI clinics performed nucleic acid amplification tests (NAATs). Ternopil and Dnipropetrovsk regional STI clinics reported AMR testing through participation in a collaborative study of WHO Collaborating Centre for Gonorrhoea and other STIs, National Reference Laboratory for STIs, Örebro, Sweden (WHO CC).

In symptomatic heterosexuals, “three-site testing” (urogenital, pharyngeal, and rectal specimens) was conducted by 25% of the respondents, “two-site testing” (urogenital and pharyngeal specimens) was conducted by 41.7% of the respondents, and “one-site testing” (urogenital specimen) was conducted by 33.3% of the respondents.

Culture performance characteristics for *N. gonorrhoeae* detection are shown in Table 4. Immediate laboratory processing after sampling was performed by 50% of the STI clinics. The remaining 50% of the respondents used temporary storage and transportation of specimens to the laboratories. The average transportation time for the specimens was 6.3 hs (range: 0.75-24 h). The majority of the respondents (75%) used direct culturing method and transported inoculated tubes/Petri dishes to the laboratory setting in thermally protected boxes. Two (16.7%) STI clinics reported the usage of combined (bedside and deferred culture) method of *N. gonorrhoeae* culturing. One STI clinic (8.3%) reported a deferred culturing method using a non-nutritive Amies Agar Gel Medium with charcoal (Copan Diagnostics Inc., Brescia, Italy) for transport.

Respondents used culture media from six different international manufacturers. The majority (79.9%) of the STI clinics isolated *N. gonorrhoeae* onto a selective culture medium (Thayer Martin agar, HiMedia Laboratories Pvt. Ltd., Mumbai, India). One of the clinics used two selective media (Chocolate agar^TM^ and PolyViteX VCAT3 medium, bioMérieux, Marcy-l’Étoile, France) and a selective reagent kit (SVG, St. Petersburg, Russia) for the isolation of gonococci using the culture method. A combination of selective and non-selective culture media was used by 16.7% of the respondents. Only non-selective culture medium was used in 16.7% of the cases. Due to restricted budget, most of the clinics (58.3%) used culture media in 7-10 mL tubes. Mixed usage of culture media in the tubes and Petri dishes was reported by 25% of the respondents. Only the Petri dish was used by 16.7% of the respondents.

All respondents identified *N. gonorrhoeae* in culture by microscopic examination after Gram-staining and by oxidase test. One of the STI clinics used the catalase test additionally. Biochemical identification was available in 75% of the respondent clinics. External quality control was supported by half of the respondents (50%). Two respondents specified WHO CC as the provider of external quality control.

## Discussion

The present study provided a portrait of public laboratory management of gonorrhoea in leading regional STI clinics in Ukraine. Survey data were compared with WHO laboratory manual requirements regarding *N. gonorrhoeae* detection. The data revealed the insufficient level of testing and diagnostic strategy for gonorrhoea in Ukraine.

All STI care providers could perform microscopy, which is the cheapest and a quick method of testing.^17^ Microbial culture was used widely in regional STI clinics (85.7%) predominantly for diagnostic purposes and rarely for screening, but not for AMR testing.

Sampling and timely specimen processing play a vital role in the pre-analytical stage of *N. gonorrhoeae* culture.^17–20^ The use of transport media ensures the viability of *N. gonorrhoeae* isolates.^17,27^ The present study showed that 50% of the regional STI clinics could not perform immediate laboratory processing of the samples. However, deferred culture method was used only by 25% of the respondents.

Few laboratories (16.7%) used non-selective culture media, which could be a reason for false-negative culture results. Abundant coexisting flora from the urogenital and especially from the extragenital specimens could inhibit the growth of *N. gonorrhoeae*.^17^ The WHO laboratory manual recommends the use of Petri dishes for culture. However, 58.3% respondents used tubes for *N. gonorrhoeae* culture. Every laboratory should validate culture media for *N. gonorrhoeae* isolation before implementation in the routine usage. Reference tests should be applied for validation.^17^ However, to the best of the author’s knowledge, WHO reference strains are not available in Ukraine.

Presumptive *Neisseria spp*. identification is performed according to the WHO laboratory manual. However, 25% of the respondents did not perform biochemical identification of suspected colonies, which could be a reason for false-positive results in cases with commensal *Neisseria species*. Similarly, the absence of biochemical identification could lead to misdiagnosis of *N. meningitidis* urogenital infections in rare cases.^17^

External quality control evaluation should be established and run regularly in every laboratory. However, in the present study, only half of the respondents participated in an external quality control system. External quality control should be performed by the certified providers or by the more informal practice of sample exchange between the laboratories.^17^ Only two respondents specified WHO CC as the provider for their external evaluation program.^12,28^

NAATs have superior sensitivity and specificity and allow detection of different STIs in a single sample.^11,12,17–20^ A recent study indicated that a significant number of gonorrhoea cases were underdiagnosed by microscopy (28.6%) and culture (42.9%) when compared with NAATs in Ukraine.^12^ An effective international experience regarding the combination of NAATs and targeted deferred culture diagnostics should be implemented in Ukraine to avoid failures in the detection of gonorrhoea.^29^ Worryingly, regional STI clinics in Ukraine did not use NAATs due to restricted budget. Private clinics and/or laboratories have an opportunity to utilise NAATs in Ukraine. Unfortunately, reports on NAATs reagents and validation results of private clinics were not available for analysis in this study. It is crucial to implement validated and quality-assured NAATs in Ukraine.

In the time of limited resources, quality-assured POCT with acceptable NAATs-based validated performance characteristics could be used for screening of STIs including gonorrhoea.^30–32^ Essentially, validation tests should be performed before the introduction of POCT into routine practice.^17^

It is important to ensure increased access to culture and antimicrobial susceptibility testing in the era of increasing spread of AMR in *N. gonorrhoeae*.^17^ A national antimicrobial susceptibility surveillance programme does not exist in Ukraine and only two regions participated in recent a pilot study.^28,33^

The WHO laboratory manual recommends sampling of urogenital specimens along with pharyngeal and rectal specimens if indicated.^17^ Rectal and pharyngeal gonorrhoea is exceedingly common. Limited usage of “three-site testing” may result in a substantial risk of missed gonorrhoea cases in Europe^22,23^ and especially in Ukraine.^34^

The main limitation of the present study was that no data from urology, gynaecology clinics and private medical centres/laboratories were collected. However, regional STI clinics, as leading STI healthcare providers, could accurately represent the current Ukrainian diagnostic management of gonorrhoea. In future Ukrainian surveys, it would be valuable to collect data from all STI providers. Additional information about sexual partners, patients’ sexual behaviour, way of sexual transmission, possible countries where gonorrhoea was acquired, treatment details, patients’ follow-up data, complicated gonorrhoea cases and treatment failures will be important.

In conclusion, the first extended assessment of laboratory diagnostics for gonorrhoea in Ukraine and their comparison with the WHO standards were presented. Increased adherence of national guidelines to the evidence-based WHO laboratory manual regarding diagnosis of gonorrhoea is essential for monitoring of gonorrhoea and preventing AMR of *N. gonorrhoeae* in Ukraine. Diagnostic management of gonococcal infections across Ukraine is insufficient and needs improvement through implementation of validated and quality-assured cultures, AMR testing, NAATs, POCT, “three-site testing”, and external quality control in regional STI clinics.

**Main Points**

- Timely monitoring of national management strategy for *N. gonorrhoaea* infection is an essential tool to improve the diagnostics of gonococcal infection.
- Evidence-based WHO laboratory guidelines should be a reference for an audit of *N. gonorrhoaea* diagnostics.
- A survey is a quick method of obtaining data regarding existing management of *N. gonorrhoaea* in a large territory and is helpful in determining key challenges for improvement.
- Extensive studies in the future that include all medical STI providers such as gynaecologists, urologists, and private medical care will be extremely valuable.

## Figures and Tables

**Table 1.** Responding Rate of Regional STI Clinics Participated in the Survey Regarding the Diagnostics of Gonorrhoea in Ukraine

Regions of STI clinics	Regions (n)	Respondents (n)	Respondents (%)
Western regions (Ivano-Frankivsk^b^, Ternopil^a^, Volyn^a^, Chernivtsi^b^, Khmelnitsky^b^)	8	5	62.5
Central regions (Kirovograd^a^, Poltava^b^)	5	2	40.0
Eastern regions (Kharkiv^c^)	3	1	33.3
Northern regions (Zhytomyr^b^, Sumy^b^, Chernihiv^b^)	4	3	75
Southern regions (Odessa^c^, Mykolaiv^b^, Dnipropetrovsk^c^)	7	3	42.9
Total	27	14	51.9

a A city with 200 000-250 000 population.

b A city with 250 000-1 000 000 population.

c A city with over 1 000 000 population. n absolute number; STI, sexually transmitted infections.

**Table 2.** Positive Results of Neisseria Gonorrhoeae Testing by Microscopy and Culture in Regional STI Clinics, Ukraine

Respondents of regional STI clinic	Microscopy				Culture			
	Tested patients (n)	Positive result			Tested patients (n)	Positive results		
		n	%	(95CI)		n	%	(95CI)
Volyn	3304	24	0.7	(0.4-1.1)	362	6	1.7	(0.6-3.6)
Kharkiv	2020	19	0.9	(0.5-1.4)	1542	7	0.5	(0.2-1)
Chernivtsi	8993	133	1.5	(1.3-1.8)	5776	80	1.4	(1.1-1.7)
Kirovograd	3024	48	1.6	(1.2-2.1)	3024	48	1.6	(1.2-2.1)
Ternopil	5523	92	1.7	(1.4-2.1)	2400	76	3.2	(2.5-4)
Poltava	5114	89	1.7	(1.4-2.1)	1516	4	0.3	(0.1-0.7)
Ivano-Frankivsk	2280	48	2.1	(1.6-2.8)	194	13	6.7	(3.6-11.2)
Mykolaiv	7203	152	2.1	(1.8-2.5)	7203	152	2.1	(1.8-2.5)
Odessa	9169	213	2.3	(2-2.6)	9169	213	2.3	(2-2.6)
Dnipropetrovsk	13,379	339	2.5	(2.2-2.8)	13,379	234	1.7	(1.5-1.9)
Zhytomyr	2179	58	2.7	(2.1-3.5)	2179	58	2.7	(2.1-3.5)
Khmelnitsky	3922	115	2.9	(2.4-3.5)	2814	73	2.6	(2-3.3)
Sumy	7882	239	3.0	(2.6-3.4)	4376	227	5.2	(4.6-5.9)
Chernihiv	2386	171	7.2	(6.2-8.3)	2339	131	5.6	(4.7-6.6)
Total	76,378	1740	2.3	(2.2-2.4)	56,273	1322	2.3	(2.2-2.4)

CI: confidence interval, n: absolute number, STI: sexually transmitted infection

**Table 3.** Diagnostic Management of Neisseria Gonorrhoeae Infections in Regional STI Clinics, Ukraine

Diagnostic characteristics		n	%	(95CI)
Access to diagnostic methods, n = 14	Microscopy	14	100	(76.8-100)
	Culture	12	85.7	(57.2-98.2)
	POCT	1	7.1	(0.2-33.8)
Antimicrobial resistance tests, n = 14		2	14.3	(1.8-42.8)
Tested sites in symptomatic heterosexuals, n = 12	1 site (urogenital)	4	33.3	(9.9-65.1)
	2 sites (urogenital and pharyngeal)	5	41.7	(15.2-72.4)
	3 sites (urogenital, pharyngeal and rectal)	3	25	(5.5-57.2)

CI, confidence interval; n, absolute number; POCT, point-of-care-test.

**Table 4.** Performance Characteristics of Neisseria Gonorrhoeae Culture in Regional STI Clinics, Ukraine

Culture characteristic, n = 12		n	%	(95CI)
Mode of inoculation	Direct	9	75	(42.8-94.5)
	Deferred	1	8.3	(0.2-38.4)
	Direct and deferred	2	16.7	(2.1-48.5)
Laboratory resource for culture	Tubes	7	58.3	(27.6-84.8)
	Dishes	2	16.7	(2.1-48.5)
	Tubes and dishes	3	25	(5.5-57.2)
Identifications test for culture	Microscopy	12	100	(73.5-100)
	Oxidase test	12	100	(73.5-100)
	Catalase test	1	8.3	(0.2-38.4)
	Biochemical	9	75	(42.8-94.5)
External quality evaluation control		6	50	(21.1-78.9)

CI, confidence interval; n, absolute number.
